# SMS photograph-based external quality assessment of reading and interpretation of malaria rapid diagnostic tests in the Democratic Republic of the Congo

**DOI:** 10.1186/s12936-014-0535-9

**Published:** 2015-01-28

**Authors:** Pierre Mukadi, Philippe Gillet, Barbara Barbé, Jean Luamba, Albert Lukuka, Joris Likwela, Dieudonné Mumba, Jean-Jacques Muyembe, Pascal Lutumba, Jan Jacobs

**Affiliations:** National Institute of Biomedical Research, Kinshasa, Democratic Republic of the Congo; National Pedagogic University, Kinshasa, Democratic Republic of the Congo; Institute of Tropical Medicine, Department of Clinical Sciences, Antwerp, Belgium; National Malaria Control Programme, Kinshasa, Democratic Republic of the Congo; University of Kinshasa, Kinshasa, Democratic Republic of the Congo; University of Lubumbashi, Lubumbashi, Democratic Republic of the Congo; Catholic University of Leuven, Faculty of Medicine, Department of Microbiology and Immunology, Leuven, Belgium

**Keywords:** Malaria RDT, External quality assessment, Short message service, DRC

## Abstract

**Background:**

The present External Quality Assessment (EQA) assessed reading and interpretation of malaria rapid diagnostic tests (RDTs) in the Democratic Republic of the Congo (DRC).

**Methods:**

The EQA consisted of (i) 10 high-resolution printed photographs displaying cassettes with real-life results and multiple choice questions (MCQ) addressing individual health workers (HW), and (ii) a questionnaire on RDT use addressing the laboratory of health facilities (HF). Answers were transmitted through short message services (SMS).

**Results:**

The EQA comprised 2344 HW and 1028 HF covering 10/11 provinces in DRC. Overall, median HW score (sum of correct answers on 10 MCQ photographs for each HW) was 9.0 (interquartile range 7.5 – 10); MCQ scores (the % of correct answers for a particular photograph) ranged from 54.8% to 91.6%. Most common errors were (i) reading or interpreting faint or weak line intensities as negative (3.3%, 7.2%, 24.3% and 29.1% for 4 MCQ photographs), (ii) failure to distinguish the correct *Plasmodium* species (3.4% to 7.0%), (iii) missing invalid test results (8.4% and 23.6%) and (iv) missing negative test results (10.0% and 12.4%). HW who were trained less than 12 months ago had best MCQ scores for 7/10 photographs as well as a significantly higher proportion of 10/10 scores, but absolute differences in MCQ scores were small. HW who had participated in a previous EQA performed significantly better for 4/10 photographs compared to those who had not. Except for two photographs, MCQ scores were comparable for all levels of the HF hierarchy and non-laboratory staff (HW from health posts) had similar performance as to laboratory staff. Main findings of the questionnaire were (i) use of other RDT products than recommended by the national malaria control programme (nearly 20% of participating HF), (ii) lack of training for a third (33.6%) of HF, (iii) high proportions (two-thirds, 66.5%) of HF reporting stock-outs.

**Conclusions:**

The present EQA revealed common errors in RDT reading and interpretation by HW in DRC. Performances of non-laboratory and laboratory staff were similar and dedicated training was shown to improve HW competence although to a moderate extent. Problems in supply, distribution and training of RDTs were detected.

## Background

Rapid diagnostic tests (RDTs) are increasingly rolled out as a tool for malaria diagnosis in malaria endemic countries. They are accurate and easy to use, requiring only a minimum of training [1,2]. Although robust and simple to perform, they remain subject to errors, part of which is related to reading and interpretation [3-5].

Performance of RDTs by end-users is not easy to measure. External quality assessments (EQA, also referred to as “proficiency testing”), in which an authorized organization sends out samples for analysis, assess the competence of diagnostic laboratories and may generate additional information, for instance about errors in the instructions for use (IFU) RDTs [6].

In the Democratic Republic of the Congo (DRC), a Central African country with one of the highest malaria burdens in the world [7,8], malaria RDTs have been deployed by the National Malaria Control Programme (Programme National de Lutte contre le Paludisme, PNLP) since 2010. The RDT product selected and diffused is the three-band SD BIOLINE Malaria Ag *Pf*/Pan HRP2/pLDH (Standard Diagnostics, Inc, Kyonggi-do, Korea), the HRP-2 line is specific for *Plasmodium falciparum*, whereas the pan-pLDH line detects all *Plasmodium* species [9].

In 2012, an EQA addressing correct reading and interpretation of RDTs was organized among laboratory health workers (HW) in DRC [3]. High-resolution photographs of RDTs with different test and control line combinations were sent out to diagnostic laboratories and the individual health workers (HW) replied by a Short Message Service (SMS). Based on the results of this EQA, training materials and job aids were adapted and updated, and were subsequently used during trainings organized by PNLP.

In October 2013, a second photograph and SMS-based EQA on reading and interpretation of RDTs by HW in DRC was organized. In addition, a questionnaire about RDT testing was supplied to the participating laboratories. The objectives of the EQA photographs were (i) to assess the competence of the individual HW in reading and interpretation of malaria RDTs, (ii) to identify common reading and interpretation errors, and (iii) to compare the results of HW who were recently trained in RDTuse and HW who had participated in the EQA in 2012 (further referred to as EQA-2012) versus HW who had not been trained and had not participated in the EQA-2012.

Additional objectives were (iv) to compare the results of the EQA photographs between HW of different provinces and (v) between HW working at different levels of the health facility (HF) hierarchy (from health post to hospital reference laboratory). The objectives of the questionnaire were to obtain information on (i) RDT products (brands) used, (ii) training on and experience with RDT use, (iii) numbers of RDTs performed and positivity rates as well as (iv) RDT supply.

## Methods

### Design

The EQA was performed from October 2013 till March 2014 in 10 of 11 provinces in DRC. The materials comprised (i) one set of photographs with multiple choice questions (MCQ) to be filled in and replied by the individual HW and (ii) one standard EQA questionnaire addressing RDT diagnostic practices with open questions and questions in multiple choice format to be filled in by the laboratory supervisor or, in case of health post, the HW in charge. In addition, printed explanations and instructions were supplied. The photographs and questionnaires were hand-delivered on site by co-investigators who explained the EQA and asked for consent. The answers – both to the photographs and the questionnaire – were sent by SMS to the study coordinator.

### Photographs

**Figure 1:**
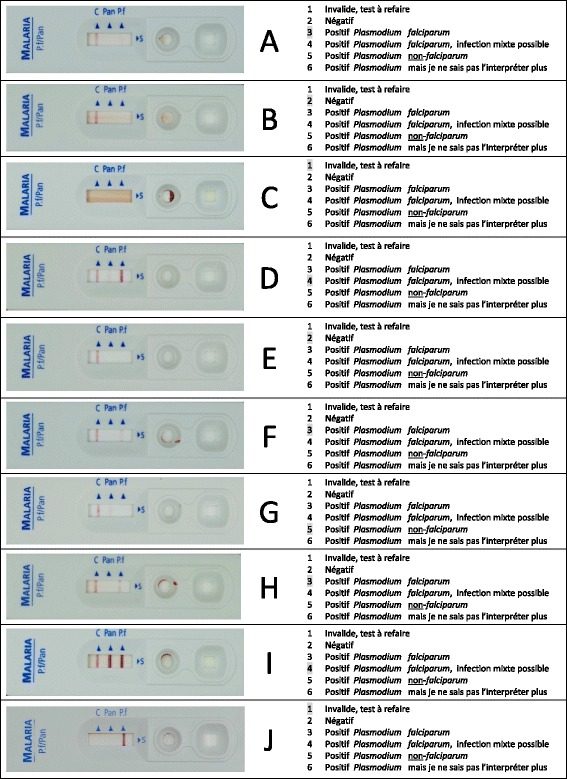
**Photograph with multiple choice questions of 10 SD malaria Ag Pf/Pan RDT results as presented to the participants.** The photographs **A** – **J** (left) represent real-life results of the SD malaria Ag Pf/Pan rapid diagnostic test; at the right hand, the options of the multiple choice questions corresponding to each of the photographs are listed. The correct answer (option) for each photograph is grey-highlighted, the details for control and test lines are listed in Table 1.

High-resolution photographs printed on glossy photographic paper (maco silk normal full colour CMYK, 300dpi, Bulckens, Herenthout, Belgium) were supplied; they depicted cassettes of the RDT recommended and distributed by the PNLP, the SD malaria Ag Pf/Pan (HRP2/ pLDH, Standard Diagnostics, Inc., Kyonggi-do, Korea) with different combinations and intensities of control and test lines in real-life dimensions (Figure 1). SD Bioline malaria Ag/Pan is a so-called three-band RDT (one control and two test lines) detecting Histidine-Rich Protein 2 (HRP-2) and pan-*Plasmodium* lactate dehydrogenase (pan-pLDH).

The photographs were identical to those used during EQA-2012 (with the order being changed), apart from one: the invalid test photograph displaying a non-cleared background was replaced by another invalid test photograph displaying a *P. falciparum* (Pf) test line in the absence of a control line. Photographs presented combinations of invalid and negative results, positive faint and weak test lines, and combinations of different *Plasmodium* species. The MCQ options (n = 6) were listed on the right hand side of the photographs, and were numbered from 1 to 6. Only one option per MCQ question was considered correct. Photographs had been validated through an expert panel and through a previous EQA [3]. In line with EQA-2012, the SMS reply contained the following information (i) the code “Eeq” (for “Evaluation externe de la qualité” or EQA), (ii) a 10 digit answers code consisting of the combination of the options (number from 1 to 6) for each of the 10 MCQs, (iii) the participant’s name, (iv) the HF of the HW and (v) the name of the province. Participants were transferred 1US$ of phone credit. After closing of the EQA, an SMS message with the correct answer for the MCQs was sent to each EQA participant.

### Questionnaire

The questionnaire addressed the current practices of the participating HF with regard to RDT product used, training and experience, numbers of test performed and positivity rate, and supply. In addition, participants were asked if they had been trained. The questionnaire comprised MCQs (n = 10) with 2, 3, 4, 5 or 6 options (numbered from 1 to 6) and open answer questions (n = 2) to be replied as numbers with three digits (example “100”). Answers were sent to the study coordinator by SMS, containing the following information (i) the code “Qes” (for “Questionnaire”), (ii) a 16 digit answer code consisting of the combination of the options and the names of (iii) laboratory supervisor, (iv) HF, and (v) province.

### Participating health facilities and health workers

HF were selected from 10 out of 11 provinces in DRC by the co-investigators based on accessibility and travel opportunities (for instance, during supervision visits or trainings), thereby assuring an equal representation of the different levels of the HF hierarchy. The HF were part of the networks of the National Tuberculosis Control Program (PNLT) or of the National Institute of Biomedical Research (INRB). The Province of Nord Kivu was not included because of security reasons related to the on-going war. In practice, HW were addressed through the laboratory supervisor and comprised laboratory-trained staff, except for the health posts, which were staffed by medically trained non-laboratory staff. As recommended by PNLP, in HF where microscopy is available – in practice hospitals and reference health centers, microscopy is preferred over RDTs for malaria diagnosis, whereas health centers and health posts rely on RDTs. A total of 2,500 HW and 1,000 HF were targeted, in practice 2,550 photograph prints had been sent out.

### EQA sample and result flow

Envelopes containing photographs, questionnaire, instructions and informed consent form for the questionnaire were shipped by private air carrier to the provincial airports where they were received by the provincial co-investigators (n = 14), which were staff members from the provincial sections of PNLP and PNLT or representatives of the Provincial Division of the Ministry of Health. For Kinshasa and eastern part of Bas-Congo province, the INRB collaborators and PNLT co-investigator transported the envelopes by car and motorcycle respectively.

The provincial co-investigators visited the HF in person and had a meeting with the laboratory or HF supervisor for the explanation of the objectives and the procedure of the EQA and for asking consent for the questionnaire. For the photographs, the HW were explained that by participating (*i.e.* sending the SMS answers), they agreed to give implicit consent. Next, the HW replied individually to the photograph MCQs by sending an SMS to the study coordinator. The laboratory supervisor of each HF replied to the questionnaire by sending an SMS to the study coordinator.

### Data entry and analysis

SMS answers (photographs and questionnaire) were transferred by Bluetooth to an Excel database managed by the study coordinator. SMS answers that were incomplete, had a too long code (>10 for photographs or > 16 for questionnaires) or contained numbers higher than six were considered as ineligible and were removed. In the final database, the names of the participating HW and HF were removed.

MCQ-answers were considered correct if the correct option was answered. For each individual HW, the sum of correct answers to each out of 10 MCQ represented a score on 10 (“HW score”). For each individual photograph MCQ, the proportion (%) of HW replying correctly was considered as the “MCQ score”. As to the interpretation, an incorrect answer was considered as “major error” in case the diagnosis of malaria and/or the presence of *P. falciparum* were missed. Common errors in reading and interpretation as apparent from different MCQs were grouped together.

Scores between HW who had been trained during the past 12 months (*i.e.* after EQA-2012) and who had participated in EQA-2012 were compared to the scores of HW who had not been trained recently and did not participate to the EQA-2012. In addition, scores were compared between provinces and according to HF hierarchy. Differences between proportions were tested for significance using the Chi-square or, in case of small sample sizes a two-tailed Fisher’s exact test. Differences in the mean and median values were assessed for significance by the Student’s *t*-test. A p-value < 0.05 was considered significant.

### Ethics statement

The study protocol was approved by the Institutional Review Board (IRB) of the Institute of Tropical Medicine (ITM) and the Ethical Committee of Antwerp University (IRB/AB/ac/093, Ref: 887/13 of 5/6/2013). The participation was voluntary and no pressure during on-site visits had been exerted.

There was implicit and written consent for participating to the photograph MCQ and questionnaire respectively. The identity of the HW was known only to the principal investigator and was not shared with PNLP nor with any other organization.

## Results

### Participating health workers and health facilities

The EQA was sent out on October 22th 2013 and was closed after 21 weeks; a reminder was done (by telephone) at week 9. Delays in distribution of EQA materials occurred in the Provinces of Maniema and Sud-Kivu. At the time of the planned closing date (*i.e.* eight weeks after start of shipment), 2,039 SMS (79.9% of distributed photographs) were received, and reminders were sent. At the closure, a total of 2,349 non duplicate SMS were received for the photograph MCQs (2,161 by phone and an additional 188 by email from areas which were not covered by phone network). After removal of five non-eligible SMS, 2,344 answers from 1,028 HF were eligible, corresponding to 91.9% (2,344/2,550) of shipped photographs.

For the questionnaire, 1,043 non duplicate SMS replies were received. After removal of 15 ineligible SMS, 1,028 answers from HF were eligible, corresponding to 98.6% (1,028/1,043) of distributed questionnaires. About half (n = 575, 55.9%) of the 1,028 participating HF belonged to the “Health Centre” level, comprising 47.8% (1121/2344) of HW. The “Health Post” level (staffed with non-laboratory professionals) represented 4.4% (n = 104) of HF and 5.0% (n = 118) of HW. The category “Other HF” (33 (3.2%) of HF and 214 (9.1%) of HW) represented private laboratories and training institutes for laboratory staff.

**Figure 2:**
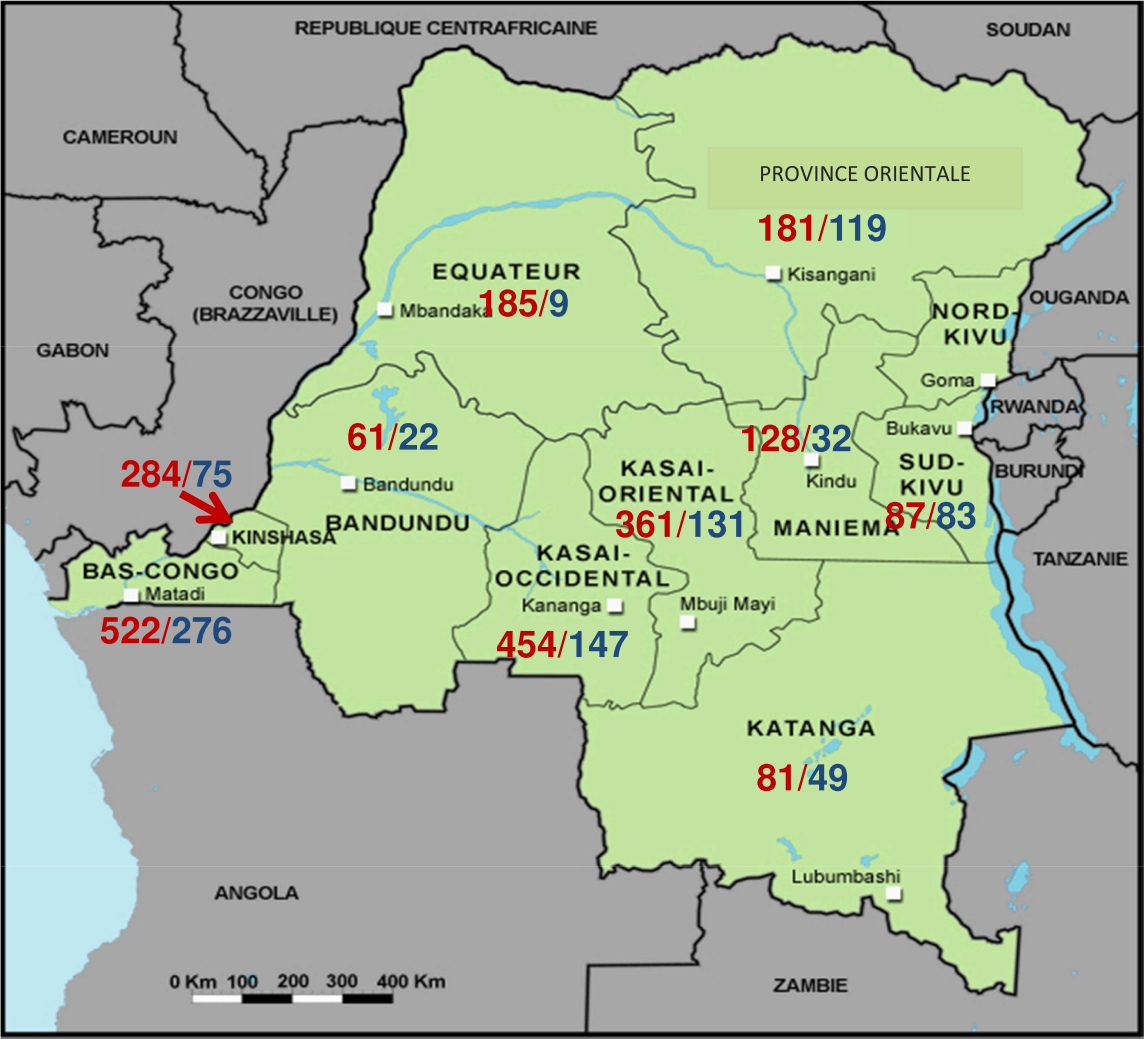
**Geographical distribution of participants of the external quality assessment.** The map represents the 11 provinces of the Democratic Republic of the Congo. Data represent numbers of Health workers (red)/Health facilities (blue).

Figure 2 shows the geographic distribution of HW and HF. Most HW and HF (41.6% and 41.1% respectively) were from the provinces of Bas-Congo and Kasaï Occidental.

**Figure 3:**
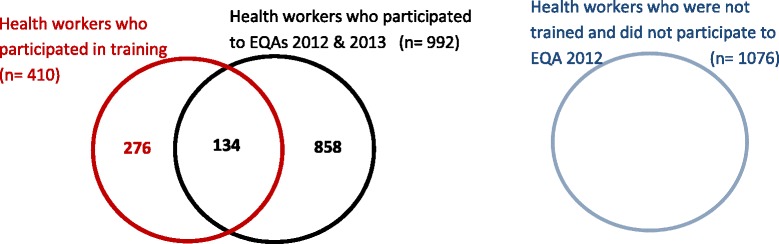
**Different groups of participants of the external quality assessment (EQA).**

Figure 3 shows the distribution of HW according to their participation to EQA-2012 and the training they received < 12 months ago.

### Photographs presented as multiple choice questions to the individual health workers

The photographs with MCQ as presented to the HW are depicted in Figure 1.

### Overall performances: HW scores and photograph MCQ scores

Table 1 displays the results of the answers for each photograph MCQ. The median HW score was 9.0 (IQR 7.5 – 10), with 37.8% (886/2344) of HW reaching a score of 10. The MCQ scores ranged from 54.8% to 91.6%. Lowest scores (<70%) were for photograph A presenting a faint *P. falciparum* test line and for photograph D presenting a faint pan-pLDH test line.

**Table 1:** **Composition of the external quality assessment and scores for each photograph and multiple choice question (MCQ-scores)**

**Photograph**	**Lines present**			**Possible options given for the multiple choice questions**						**Comments**
	**C**	**Pan**	***P.f.***	**1. Invalid**	**2. Negative**	**3. Positive** ***P.f.***	**4. Positive,** ***P.f.*** **mixed infection possible**	**5. Positive,** ***P.*** **non** ***- falciparum***	**6. Positive, but species not known**	
A	+	-	F	6.9	29.1	**54.8**	3.0	1.7	4.5	Faint line (*P.f.*-HRP2) intensity read or interpreted as negative: 29.1%
B	+	-	-	5.2	**90.0**	2.6	0.4	0.7	1.0	Missing negative test: 10.0%
C	-	-	-	**91.6**	6.1	0.9	0.6	0.3	0.4	Missing invalid test (no control line, no test line): 8.4%
D	+	F	S	5.1	1.5	24.3	**65.7**	1.3	2.1	Faint line (pLDH) intensity read or interpreted as negative: 24.3%
										Failure to distinguish presence of *P.f.*: 1.3% + 2.1% = 3.4%
E	+	-	-	6.5	**87.6**	2.1	1.8	1.0	1.0	Missing negative test: 12.4%
F	+	-	W	3.9	3.3	**82.8**	3.0	3.2	3.8	Failure to distinguish presence of *P.f.*: 3.2% + 3.8% = 7.0%
										Incorrect reading as negative: 3.3%
G	+	F	-	6.4	7.2	6.1	4.7	**70.7**	4.9	Faint line (Pan-pLDH) intensity read as negative = 7.2%
										Wrongly interpreted as *P.f.:* 6.1% + 4.7% = 10.8%
H	+	-	M	3.5	1.5	**85.9**	4.4	2.9	1.8	Medium line (*P.f.-*HRP2) intensity read or interpreted as negative = 1.5%
										Failure to distinguish presence of *P.f.*: 2.9% + 1.8% = 4.7%
I	+	S	S	2.0	0.6	4.1	**89.9**	1.1	2.3	Failure to distinguish presence of *P.f.*: 1.1% + 2.3% = 3.4%
J	-	-	S	**76.4**	6.6	7.4	1.0	1.0	7.6	Missing invalid test; No control line, presence of test line: 23.6%

The MCQ-scores present the percentage of health workers (n = 2344) who correctly replied. See Figure 1 for the picture of the photographs. Correct answers are displayed in bold.

*Abbreviations* and symbols: *C* control line, *Pan* *Plasmodium* spp. (all four species), *P.f.* Plasmodium falciparum, *−* negative (no line present), *+* positive (visible control line present), *F* faint positive line intensity compared to the control line, *M* medium positive line intensity compared to the control line, *W* weak positive line intensity compared to the control line, *S* strong positive line intensity compared to the control line.

### Common errors in reading and interpretation

Common errors observed were the following: (i) reading or interpreting faint or weak line intensities as negative, (ii) failure to distinguish the correct *Plasmodium* species, (iii) missing invalid results and (iv) missing negative results (Table 1).

(i) Faint or weak line intensities were displayed in photographs A, D, F and G, and were read or interpreted as negative by respectively 29.1%, 24.3%, 3.3% and 7.2% of HW: in these cases the non-correct answers were considered as major errors (missing the diagnosis of malaria or *P. falciparum*). (ii) A proportion of 3.4%, 7.0%, 4.7% and 3.4% of HW failed to distinguish the correct *Plasmodium* species in photographs D, F, H and I: in all four cases, the diagnosis of *P. falciparum* as the species causing malaria was missed, representing a major error. In addition, the faint line intensity for the pan-pLDH line next to a strong Pf-HRP2 line in photograph D was overlooked by 24.3% of HW.

(iii) Invalid test results were missed in photographs C and J. Of note, this error occurred more frequently in the case of photograph J (553/2344, 23.6%) – which presented a visible test line in combination with an absent control line – as compared to photograph C (197/2344, 8.4%), which showed neither control nor test lines (p < 0.001). (iv) Negative test results (photographs B and E) were missed by 10.0% and 12.4% of HW respectively. In both cases, more than half of HW who did not provide the correct answer, replied “invalid”.

### Scores for HW who had received recent training and those who had not been trained

Comparing the three groups of HW (those who were trained < 12 months ago, those who had participated in EQA-2012 and the group of “other HW” (who had neither been trained nor had participated in EQA-2012)) revealed the following. First, HW scores between the three groups did not differ much: median scores (IQR) for the three groups were 9.0 (8.0 – 10) for recently trained HW as well as for HW who had participated in EQA-2012, and 9.0 (7.0 – 10) for the group of other HW. Further, differences between MCQ-scores among the three HW groups were relatively small, they ranged from 0.0% to 9.2% and were < 5% for four photographs. However, among HW who were recently trained, the proportion with a score of 10 was higher as compared to the two other groups (48.4% versus 35.9% and 35.7%, p < 0.001). Recently trained HW had less errors for 7/10 photographs, of which five photographs had significantly better MCQ scores compared to the group of other HW (Table 2). HW who had participated in EQA-2012 performed significantly better than the “other HW group” for 4/10 photographs. A total of 134 HW had participated in EQA-2012 and had been recently trained as well (Figure 3), their scores were similar to (but not better than) those of recently trained HWs.

**Table 2:** **Common errors by health workers (HW) participating to the external quality assessment according to different groups: those who also participated to the EQA in 2012 (EQA-2012), those who were recently trained (<12 months ago), and the other HW (who had neither recently been trained nor had participated to EQA-2012)**

**Type of error**	**Photograph**	**Line**			**HW who participated to EQA-2012**	**HW trained < 12 months ago**	**Other HW**
		**C**	**Pan**	***P.f.***	**(n = 992)**	**(n = 410)**	**(n = 1076)**
Faint and weak lines overlooked	A	+	-	F	31.3	24.6	29.1
	D	+	F	S	22.9	22.2	26.6
	F	+	-	W	2.7	2.7	4.0
	H	+	F	-	5.7	4.1	9.6
Failure to distinguish the correct *Plasmodium* species	D	+	F	S	2.2	3.9	4.1
	F	+	-	W	7.1	5.6	7.2
	H	+	-	M	3.4	3.7	4.6
	I	+	S	S	2.0	3.7	4.6
Missing negative test	B	+	-	-	10.0	8.0	10.3
	E	+	-	-	13.2	9.0	12.5
Missing invalid test	C	-	-	-	6.6	11.0	9.6
	J	-	-	S	22.3	19.0	27.0

The proportions present the % of HW who made errors.

*Abbreviations* and symbols: *C* control line, *Pan* *Plasmodium* spp. (all four species), *P.f.* Plasmodium falciparum, *−* negative (no line present), *+* positive (visible control line present), F faint positive line intensity compared to the control line, *M* medium positive line intensity compared to the control line, *W* weak positive line intensity compared to the control line, *S* strong positive line intensity compared to the control line, *HW* health worker, *EQA* external quality assessment.

### Scores according to provinces of DRC and health facility hierarchy

Table 3 presents common errors by HW participating to the EQA according to the 10 participating provinces of DRC. The MCQ errors for the provinces of Bandundu and Kinshasa were under average for all 10 photographs. The MCQ scores of HW from the provinces Kinshasa and Bandundu were significantly better than those for HW from other provinces combined, as were the proportion of the HW reaching a 10/10 HW score (60.7% and 69.4% for Bandundu and Kinshasa versus 32.6% for all other provinces combined, p < 0.001).

**Table 3:** **Common errors by HW participating by HW participating to the external quality assessment according to different provinces of DRC**

**Type of error**	**Photograph**	**Line**			**Bandundu**	**Kinshasa**	**Bas-Congo**	**Equateur**	**K. Occidental**	**K. Oriental**	**Katanga**	**Maniema**	**Province Orientale**	**Sud Kivu**
		**C**	**Pan**	***P.f.***	**(n = 61)**	**(n = 284)**	**(n = 522)**	**(n = 185)**	**(n = 454)**	**(n = 361)**	**(n = 81)**	**(n = 128)**	**(n = 181)**	**(n = 87)**
Faint and weak lines overlooked	A	+	-	F	24.6	16.5	38.7	28.6	22.9	29.6	32.1	28.9	34.3	32.2
	D	+	F	S	8.2	15.5	19.5	18.4	13.9	44.3	25.9	42.2	30.9	35.6
	F	+	-	W	1.6	0.0	2.3	3.2	1.5	6.1	6.2	5.5	8.8	2.3
	H	+	F	-	1.6	0.0	0.8	2.2	2.0	1.1	1.2	2.3	5.0	0.0
Failure to distinguish the correct *Plasmodium* species	D	+	F	S	0.0	2.5	2.7	3.2	2.2	4.7	13.6	1.6	3.9	6.9
	F	+	-	W	0.0	0.4	4.4	9.2	6.2	15.0	7.4	12.5	6.1	10.3
	H	+	-	M	0.0	0.7	3.8	7.6	4.8	5.0	9.9	10.9	2.8	9.2
	I	+	S	S	0.0	0.7	4.2	4.3	1.8	2.2	7.4	13.3	3.9	2.3
Missing negative test	B	+	-	-	0.0	3.2	7.5	5.9	15.4	14.7	7.4	18.0	6.6	12.6
	E	+	-	-	3.3	3.9	7.1	8.1	17.6	19.4	8.6	21.9	18.2	9.2
Missing invalid test	C	-	-	-	4,9	1.4	13.4	5.4	8.4	7.8	4.9	12.5	10.5	5.7
	J	-	-	S	6,6	8.5	25.5	20.5	30.8	17.2	9.9	55.5	18.8	44.8

The proportions present the % of HW who made errors.

*Abbreviations* and symbols: *C* control line, *Pan* *Plasmodium* spp. (all four species), *P.f.* Plasmodium falciparum, *−* negative (no line present), *+* positive (visible control line present), *F* faint positive line intensity compared to the control line, *M* medium positive line intensity compared to the control line, *W* weak positive line intensity compared to the control line, *S* strong positive line intensity compared to the control line.

Table 4 displays the correct MCQ errors for HW from different levels of the HF hierarchy. HW from private laboratories and training institutes (“Other HF”) consequently had fewer errors for six photographs. Among HW from referral hospitals, referral health centers, health centers and health posts, MCQ scores were comparable except for two photographs (A and J). For photograph A (reading and interpreting a faint *P. falciparum*-line as positive), HW from health posts had a poor MCQ score (23.7%) which was significantly lower (p < 0.001) compared to any other HF level.

**Table 4:** **Common errors by HW participating to the external quality assessment according to the health facility (HF) hierarchy**

**Type of error**	**Photograph**	**Line**			**Referral Hospital** ^**a**^	**Referral Health Center**	**Health Center**	**Health Post**	**Other HF** ^**b**^
		**C**	**Pan**	***P.f.***	**(n = 643)**	**(n = 248)**	**(n = 1121)**	**(n = 118)**	**(n = 214)**
Faint and weak lines overlooked	A	+	-	F	23.5	29.0	31.3	62.7	15.4
	D	+	F	S	20.1	26.6	29.3	21.2	9.8
	F	+	-	W	2.8	4.4	3.9	1.7	1.4
	G	+	F	-	5.8	8.9	7.3	7.6	8.9
Failure to distinguish th correct *Plasmodium* species	D	+	F	S	3.4	3.6	3.7	4.2	1.4
	F	+	-	W	4.5	7.7	9.1	6.8	3.3
	H	+	-	M	3.7	7.7	4.9	7.6	1.9
	I	+	S	S	3.3	4.0	4.1	0.8	0.9
Missing negative test	B	+	-	-	9.0	8.1	10.7	8.5	12.1
	E	+	-	-	12.9	9.3	13.7	8.5	9.8
Missing invalid test	C	-	-	-	6.8	6.0	10.8	5.1	5.1
	J		-	S	16.0	32.3	27.0	27.1	16.4

The proportions present the % of HW who made errors.

*Abbreviations* and symbols: *C* control line, *Pan* *Plasmodium* spp. (all four species), *P.f.* Plasmodium falciparum, *−* negative (no line present), *+* positive (visible control line present), *F* faint positive line intensity compared to the control line, *M* medium positive line intensity compared to the control line, *W* weak positive line intensity compared to the control line, *S* strong positive line intensity compared to the control line.

^a^Included 2 HW from provincial reference laboratories.

^b^Other HF included private laboratories and training institutes.

### Questionnaire about RDT use among health facilities

#### RDT products used

A total of 917/1,028 (89.2%) of HF declared to be using RDTs at the moment of the EQA and/or during the year 2013. The RDT product recommended by PNLP - SD Bioline malaria Ag Pf/Pan - was used by the majority of HFs (722/917, 78.7%). Of note, 70.6% (12/17), 39.7% (54/136) and 36.6% (30/82) of HF in the provinces of Katanga, Kasaï Occidental and Equateur used another RDT product. Two two-band Pf-HRP2 products (SD Malaria antigen Pf and Paracheck Pf-Rapid Test) were used respectively by 3.4% (31/917) and 7.5% (69/917) of HF. In addition, 6.9% (64/917) of HF used the SD Bioline Pf-HRP2/*P. vivax* pLDH product, nearly two-thirds (64.1%, 41/64) of them were from the Kasaï Occidental province and represented one third (30.1%, 41/136) of the participating HF from that province.

#### Training on and experience with RDT use

About one third (308/917, 33.6%) of HF which actually used RDTs were doing so for more than two years; those who used RDTs since 1 – 12 months and since 12 – 24 months represented 25.7% (236/917) and 33.4% (306/917) of HF respectively. Overall, one third (309/917, 33.7%) of HF were actually using malaria RDTs but were never formally trained. The proportions of HF which had been trained according to HF hierarchy were 54.9%, 54.1%, 69.5% and 83.0% among referral hospitals (84/153), referral health centres (59/109), health centres (370/532) and health posts (83/100) respectively.

#### Numbers of RDT tests performed and positivity rate

In the month preceding the EQA, the HF had performed a median of 39 RDTs: nearly three-quarters (72.5%, 542/748 of eligible answers) of HF had performed less than 100 RDTs, 42.4% (317/748) of HF had performed less than 50 RDT tests. The remaining HF had performed between 101 and 300 RDTs (163/748, 21.8%) or more than 300 RDTs (43/748, 5.7%). Overall median (IQR) RDT positivity rate was 39% (IQR 0-86%); reported RDT positivity rates varied widely between < 10% (20.6% of 748 HF), 10 – 25% (9.5%), 25 – 50% (34.1%) and > 50% (35.8%). There were no apparent differences according to provinces or HF hierarchy.

#### RDT product supply

The actual stock of individual RDT tests available in the HF at the moment of the EQA was reported as less than 25 (43.9% among 902 eligible answers), between 25 and 100 (24.1%), 100 to 250 (22.2%) or more than 250 (9.9%). When comparing the numbers of RDTs used in September 2013 to the actual stock available, 42.2% (316/748) of HF had not enough RDTs available to cover the expected monthly consumption. Overall, two-thirds (66.5%, 581/874 eligible answers) of HF reported stock-outs during the year before the EQA.

## Discussion

The present EQA succeeded a previous photograph MCQ and SMS-based EQA about reading and interpretation of RDTs, organized in 2012. Considering the results of the latter, PNLP had improved training materials to anticipate the most common errors. An additional province was included (Katanga) and higher numbers of HW and HF were reached (1849 HWs and 680 HF in 2012) with a more representative distribution over the country’s provinces and health care hierarchy level. The duration of the present EQA (five months) was longer than the EQA-2012 (two months), for reasons of difficulties in distribution in Maniema and Sud-Kivu provinces and in an attempt to increase the response rate.

Although it was not an explicit objective of the present study, it is tempting to compare the overall scores between the present and the previous EQA. Compared to EQA-2012, overall HW and MCQ scores tended to improve: for EQA-2012, median (IQR) HW scores were 8.5 (7.0 – 9.5), with 18.5% of HW obtaining a score of 10 compared to 9.0 (7.5 – 10) and 37.8% for 2013. In 2012, MCQ-scores ranged from 53.7% to 90.2%, they were lower for all but 2/9 photographs which were used in both EQAs.

Not unexpectedly, the less MCQ errors were observed among HW who had been trained < 12 months ago, *i.e.* with the updated PNLP training materials which explicitly addressed the common errors revealed by EQA-2012. Although statistically significant, differences in MCQ scores were modest in absolute values and low in comparison with a recent study in Zambia, where critical steps in RDT use increased from 87.5% before to 100% after training [5]. However, the latter study involved a confined group of 65 HW who, after training, were followed up every three months during one year - with a potential bias towards the correct results. Nevertheless, the modest improvement after training observed in the present study indicates that reflection on the didactic approach of the trainings may be considered, as well as refresher trainings planned. Likewise, HW who had participated in EQA-2012 had fewer errors than HW who were not trained nor participated in EQA-2012, but again differences were small and scores were lower than those from HW who were recently trained. Taking into account the educational (didactic) impact of EQA participation [9,10], one could have expected better scores and larger differences. Possible explanations may relate to the diffusion of the feedback report of EQA-2012, which consisted of a (i) SMS message at the closing date of the EQA (so in practice several weeks after submission of the answers by the HW) and a (ii) paper version of the final EQA report which needed to be distributed through channels of PNLP, as regular mail service is not functioning in DRC.

Among the errors detected in the present and the previous EQA [3], disregarding faint test lines as negative was a common finding, which occurred at comparable frequencies. It has been observed in other studies from Lao PDR, The Philippines and Zambia [1,5,11], in the former study it was noted to recur even in well-trained end-users. In practice, the error may occur more frequently than expected - for instance when reading in unfavourable light conditions during night shifts or by elderly readers [12] - and its impact may be high (missing the diagnosis of *P. falciparum* malaria). The second error, failure to distinguish the correct *Plasmodium* species was noted at a slightly lower frequency compared to the previous EQA (4.1%- 31.0%, [3]) as well as to a study from Sudan [13]. Failure to recognize invalid test results was a third common error. For photograph C (no control line, no test line), the error occurred at similar frequency compared to EQA-2012 (13.2%, [3]). Photograph J showed no control line but a visible Pf-test line; it was not shown in EQA-2012 and was missed as “invalid” by nearly a quarter (23.6%) of HW. In terms of RDT design and mechanism, it is an unusual and very rare case, and the chances of observing it are very rare. Finally, the failure to report negative test results occurred in similar proportions as in EQA-2012 (9.8% and 12.8%, [3]). The reasons behind are unclear: it is tempting to hypothesize that, in the scope of the EQA, HW did not expect a negative test result. Nevertheless, the error when performing RDTs is less understandable than for instance in the case of malaria microscopy whereby 33.3 and 19.0% of participants to EQA sessions reported negative samples as positive [9,14].

As to the distribution of scores among the provinces, more detailed study is required to complete the picture of RDT performance in the different provinces, but it is assumed that factors such as experience and training since the introduction of RDTs are related to this observation. Of interest, overall scores were not lower in the Katanga province, *i.e.* the province with most widespread use of other RDT brands than recommended by PNLP. As expected, HW from private laboratories and training institutes presented best MCQ scores. Subtracting them from comparison, it was clear that MCQ scores and HW scores were similar among all levels of HF hierarchy. More particular, it showed that, overall, non-laboratory staff (HW from health posts) had similar performance in RDT reading and interpretation.

The questionnaire about RDT use revealed several concerns but also considerable improvements. First, 78.7% of HF declared to use the RDT product recommended by PNLP – compared to adherence by only two-thirds of HF recorded in 2012 [3]. HF using other RDT products were geographically clustered, which suggests that the choice of the RDT product may have been donor-driven. Among the RDT products used, there were two-band products as well as a *Pf*-HRP2/*Plasmodium vivax* pLDH product, the latter being not appropriate for the Central-African setting. The co-presence of different RDT products in a country poses challenges in training and supervision to the National Malaria Control Programmes and therefore should be avoided [15]. Next, although there was still a high proportion (one third) of HF which actually used RDTs but had never been trained, this proportion was considerably lower compared to 2012 (33.7% versus nearly half, 47.9% in 2012). Of note was also the high proportion (two-thirds of HF) of RDT stock-outs reported. This is of particular concern since the HF selected for the EQA were probably among the most accessible HF in the country. Numbers of RDT tests performed per month and RDT positivity rates were in line with those recorded by EQA-2012; the overall positivity rate recorded in DRC was 67.3% [16].

The present EQA shared a number of intrinsic limitations. First, it assessed competence rather than daily performance – for instance, it is not excluded that HW of the same or neighbouring HF (such as in densely-populated Kinshasa) exchanged about the MCQ photographs and convened a common answer. This could result in an overestimation of actual performances, although the tendency to search for the correct answer might have been lower in the setting of assessing individual HW compared to HF. Related to the present EQA there were other limitations: first, only the post-analytic phase (reading and interpretation) was addressed. Indeed, bench-side controls for RDT use are currently not available, and shipment and performance of clinical samples as previously done in Europe [6] is not feasible inDRC. Likewise, in case of disregarding faint test lines as negative, it could not be distinguished whether this error was due to visual reading versus interpretation. Further, the 1 US$ incentive might have attracted HW (or occasionally other persons) who actually did not have any exposure to RDTs. Reasons of poor road infrastructure delayed shipment and distribution of the EQA materials and limited the penetration in many areas. Nationwide, the estimated coverage of the present survey comprised 6% (518/8266) of health centers and 30.8% (121/393) of referral hospitals [17]. Likewise, community health workers, as well as non-actors in the private sector, had not been addressed and, there may have been a bias as participating HF where selected by previous participation, accessibility and existing contacts, which could have had an impact on the results according to provinces and health care facility hierarchy.

Despite these limitations, the present EQA generated confirmed and extended information about errors in RDT reading and interpretation and gave feedback about the renewed and improved training materials developed by PNLP. In addition, it provided insights in the use and distribution of RDTs over the country and on the different levels of health hierarchy. More difficult to measure (and not aimed in the present study) are the implicit benefits of EQAs, *i.e.* boosting self-confidence of participants and professionals [10]. In addition, use of SMS as a tool to reply to the questionnaire proved to be satisfactory, although care should be taken not to “overload” the questionnaire in terms of length and replies to be sent.

Future directions in the design and organization of EQA may further explore mobile phone technology, *e.g.* by using smartphones. Indeed, smartphone image technology has been successfully used for microscopy and RDT reading and quality control [18-20]. By consequence, smartphone applications could assure improved communication of EQA feedback reports by sending images of the original MCQ photographs with the expected results and main messages highlighted. Given the challenges of the poor infrastructure of DRC, smartphone applications could also be considered for sending out the EQA MCQ photographs, provided adequate image quality and resolution (to depict faint test lines) as well as sufficient smartphone and network coverage. Further, as done for previous EQAs, joined shipments with other disease control programs should be encouraged (such as previously done with the HIV-AIDS, tuberculosis and sleeping sickness program in DRC). Benefits of such collaboration would not only decrease costs and increase coverage, but also strengthen the overall laboratory performance and networking [21,22].

## Conclusions

The present EQA revealed common errors in RDT reading and interpretation by HW in DRC, including (i) reading or interpreting faint or weak line intensities as negative, (ii) failure to distinguish the correct *Plasmodium* species, (iii) missing invalid test results and (iv) missing negative test results. Performances of non-laboratory and laboratory staff were similar and dedicated training was shown to improve HW competence although to a moderate extent. Problems in supply, distribution and training of RDTs were detected. The use of SMS as a tool for EQA proved to be satisfactory.

## Abbreviations

Ag: Antigen
DRC: Democratic Republic of the Congo
EQA: External quality assessment(s)
HF: Health facility
HRP2: Histidine-rich protein II
HW: Health worker
IFU: The instructions for use
INRB: Institut National de Recherche Biomédicale
ITM: Institute of Tropical Medicine
IQR: Interquartile range
IRB: Institutional Review Board
MCQ(s): Multiple choice question(s)
Pan: *Plasmodium* spp
Pf: *Plasmodium falciparum*
Pv: *Plasmodium vivax*
pLDH: *Plasmodium* lactate dehydrogenase
PNLP: Programme National de Lutte contre le Paludisme
PNLT: Programme National de Lutte contre la Tuberculose
RDT(s): Rapid Diagnostic Test(s)
SD: Standard Diagnostic
SMS: Short message services
WHO: World Health Organization
